# Phytostabilization of Phosphate Mine Wastes Used as a Store-and-Release Cover to Control Acid Mine Drainage in a Semiarid Climate

**DOI:** 10.3390/plants10050900

**Published:** 2021-04-29

**Authors:** Meryem El Berkaoui, Mariam El Adnani, Rachid Hakkou, Ahmed Ouhammou, Najib Bendaou, Abdelaziz Smouni

**Affiliations:** 1 Laboratory of Plant Physiology and Biotechnology, Laboratoire Mixte International–LMI AMIR, Research Center on Plant and Microbial Biotechnology, Biodiversity and Environment, Faculty of Sciences, Mohammed V University in Rabat, Rabat 10001, Morocco; n.bendaou@um5r.ac.ma (N.B.); a.smouni@um5r.ac.ma (A.S.); 2 Mining and Environmental Engineering Laboratory, National School of Mines of Rabat, l, av. Hadj Ahmed Cherkaoui, BP 753, Rabat 53000, Morocco; eladnani@enim.ac.ma; 3 IMED-Lab, Faculty of Sciences and Technology, Cadi Ayyad University, BP 549, Marrakech 40000, Morocco; r.hakkou@uca.ma; 4 Mining Environment & Circular Economy (EMEC) Program, Mohammed VI Polytechnic University (UM6P), Ben Guerir 43150, Morocco; 5 Laboratory of Microbial Biotechnology, Agrosciences and Environment, Faculty of Sciences-Semlalia, Cadi Ayyad University, BP 2390, Marrakech 40000, Morocco; ouhammou@uca.ac.ma

**Keywords:** mine waste, rehabilitation, phytostabilization, metals and metalloids, bioaccumulation, store-and-release cover, phosphate

## Abstract

The abandoned Kettara pyrrhotite mine, located near Marrakech, Morocco, is an acid mine drainage (AMD) producer site. A store-and-release cover system made of phosphate wastes was built to prevent water infiltration and the formation of AMD. This cover system should be vegetated with appropriate plants to ensure its long-term sustainability and allow its reintegration in the surrounding ecosystem. Several indigenous plant species were studied. The choice of plant species was based mainly on their tolerance to trace elements contained in the phosphate wastes, and their low capacity to translocate these metals to their aboveground parts in order to limit the risk of pollutants transfer along the food chain. The main metals and metalloids (As, Cd, Co, Cu, Pb, Zn, Ni, Cr) are determined in 13 dominant plants naturally colonizing the store-and-release cover and their rhizospheric soils. The results showed that the phosphate cover contained high concentrations of Cr (138.04 mg/kg), Cu (119.86 mg/kg) and Cd (10.67 mg/kg) exceeding the regulatory thresholds values (Cr > 100 mg/kg, Cu > 100 mg/kg, Cd > 3 mg/kg). The studied plants revealed no hyper-accumulation of metals and metalloids, and lower concentrations in shoots than in roots. Six species (*Plantago afra, Festuca ovina, Aizoon hispanicum, Herniaria cinerea, Echium plantagineum* and *Asphodelus tenuifolius*) have bioconcentration factors greater than 1, and weak translocation factors, identifying them as appropriate candidates for phytostabilization of the phosphate cover.

## 1. Introduction

Mining and associated mineral processing operations often generate environmental impacts on soil, water and air quality. The surface storage of mine wastes can lead to loss of biodiversity and wastes are exposed to erosion [1,2,3].

Metals and metalloids are major pollutants generated by mining activities. It is important to implement sustainable solutions to either eliminate or limit their risks on the environment. Despite high toxicity levels, most metal-contaminated sites often have a diverse flora that apparently tolerates their toxicity levels.

The study of the capacity of detoxification, immobilization or absorption of metals by these toxicity resistant floras could allow the assessment of risks associated with the potential transfer of metals within the ecosystem; additionally, it can also be used as a soil remediation tool. 

**Figure 1:**
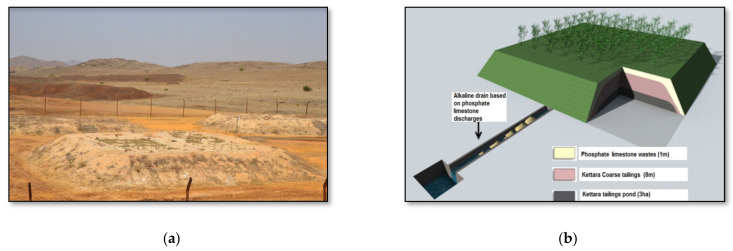
(**a**) Experimental cells for SDR cover made with phosphate mine wastes; (**b**) approved scheme for the rehabilitation of the Kettara mine site.

The abandoned Kettara mine, located in the vicinity of Marrakech, Morocco, is a good example of an area generating AMD. AMD is produced naturally when reactive sulfurous minerals in rock, such as pyrite and pyrrhotite, are exposed to water and air [4,5,6,7]. A successful reclamation design for this mine site was tested in experimental cells using a hydrogeological recovery system of the store, divert and release (SDR) type. This cover system allows the reduction of water infiltration and is considered to be one of the least expensive and most promising technologies, adapted to dry climate countries like Morocco (Figure 1). The tested SDR cover system consisted in covering the AMD generating wastes with a 1 m layer of phosphate wastes that reduced water infiltration and, effectively decreasing AMD production [8,9,10,11].

The phosphate wastes were generated by the Gantour mine, which exploits phosphate in a region near the Kettara mine. In order to finalize and to complete the proposed design, further research is needed. The establishment of a vegetation cover is necessary to stabilize the phosphate mine wastes cover and control its erosion (Figure 1). The revegetation also aims to allow the mining site to gradually return to the natural ecosystem as well as to prepare its future use. This rehabilitation is considered effective and fairly ecological and economical [12]. The choice of candidate species for revegetation is made following several criteria. First, plants must be able to grow on the phosphate waste with the least amendment possible. Secondly, they should not have a developed root system that could compromise the cover integrity. It is also necessary to choose plants with a low metal concentrations and translocation potential in order to limit the risk of pollutants transfer throughout the food chain.

Preliminary investigations were carried out on the site to inventory native species and to guide the choice of plant species to be studied for revegetation [13].

In this study, the approach is applied directly to the spontaneous vegetation of the phosphate covering by assessing and comparing concentrations of different existing metals and metalloids. The objective is to define a procedure of selection among the spontaneous plants based on their capacity of absorbing metals and metalloids in their root system, enabling thus the phytostabilization of the Kettara mining site.

## 2. Results and Discussion

### 2.1. Metal Accumulation in Phosphate Wastes

The mean total concentrations of metals and metalloids in the phosphate waste cover and their standard deviations are reported in Table 1.

In general, compared to their critical values, results show that the total concentration of Cd (10.67 mg/kg) was more than three-times higher than its critical value (3.0 mg/kg). Similarly, the Chrome (Cr) and Copper (Cu) total contents represent 138.04 mg/kg and 119.86 mg/kg, exceeding critical toxicity levels of 100 mg/kg. These results reveal that the phosphate wastes were contaminated by Cr, Cu and Cd. On the other hand, the total concentration of As, Co, Ni, Pb, and Zn were lower than their critical values, with the Co presenting the lowest percentage (1.44%) of its tolerable level. Compared to their tolerable levels, these metals concentrations vary from about 25.07% for the Ni to 53.15% for As, indicating therefore a low to moderate contamination (Table 1).

The mobile and mobilizable fractions are expressed as percentages of the extracted concentration relative to the total concentration the phosphate wastes (Table 1). Our results showed that the mobilizable fraction was consistently higher than the mobile fraction for all metals considered. In general, the mobile fraction was almost zero for many of the metals considered. The mobilizable fractions remained low, representing 0 to 2.57% of the total metal concentration, with Zn being the highest followed by Cu then Pb, Cd, As, and Co. Thus, the studied metals were not likely soluble and could therefore not be easily assimilated by plants.

The spontaneous plants that developed over the phosphate waste cover may have some tolerance to the substrate, which provided relatively favorable growing conditions. Indeed the analysis showed that the soil presented a low mean EDTA and CaCl_2_ percentage of the total As, Cd, Co, Cu, Pb and Zn concentration. These results are in accordance with previous findings [15] who studied the accumulation of metals in native plants growing on a phosphate-rich soil in Tunisia. Similar to results from the present study, they found high total concentrations of Cr and Cu in soil. They indicated that the soil was contaminated with Cr and Cu, whose mobile fraction was almost null, suggesting that their leaching was low under natural conditions. In general, the available mobile and mobilizable fractions are more indicative than the total amount of heavy metal, as these forms can predict the risk of metal uptake by plants and its mobility in the system [16]. Our results indicate that the pH value (7.53) was neutral to slightly alkaline on phosphate wastes, due to its limestone nature, which probably contributed to the immobilization of metals, and therefore presented no immediate risk to the environment. According to Hakkou et al. (2009) [17], there remained a high concentration of phosphates in the cover wastes (P_2_O_5_ 9.7–19.6 wt %). Since it is known that phosphate immobilizes Pb [18], it could explain the low concentration and availability of Pb. One approach to estimate global metal concentrations in a soil sample relies on the soil pollution index (PIs). In the present study’s results, the PIs value (0.94) was slightly less than 1, suggesting that overall, the phosphate wastes were not contaminated by metals despite a high contamination by Cd, Cu and Cr.

### 2.2. Screening of Native Plant Species in the Phosphate Wastes

The selected plant species along with their sizes and life forms are presented in Table 2. There was a total of 13 species belonging to 11 families according to the nomenclature of “Flore Pratique du Maroc” [19,20], of which 3 belong to *Asteraceae*. These plant families represent the dominant component of the natural vegetation on the phosphate cover at the Kettara mine. These species were mainly annuals (10 over 13). Plants appeared in greater numbers on the cover substrate than on the dominant edaphic conditions of the region. This observation may be related to the survival potential and characteristics of these plants, like their ability to grow quickly, survive in barren sites, and being drought resistance [21]. In addition, herb plants with fine or light seeds can be easily distributed by wind and are more likely to form heavy metal tolerance [22]. The different species showed a significant difference (*p* ≤ 0.05) between the length of the aboveground parts which ranged from 4 to 70 cm. As far as the roots, they were shallow and did not go deep into the cover. They varied between 5 and 19 cm in depth. These root sizes confirm the initial choice of plants with a poorly developed root system in order to preserve the cover integrity.

### 2.3. Heavy Metal Concentrations in Plant Tissues

Concentrations of the studied metals and metalloids in the shoots and roots of the plant species are shown in Table 3 and Table 4, respectively. The Cr and Co concentrations were significantly higher (*p* ≤ 0.05) than other metals, both in roots and shoots, in most plant samples of the phosphate cover. Statistically significant difference was observed among the concentrations of Cr in plants (*p* ≤ 0.05). Cr concentration in roots ranged from 3.08 to 49.77 mg/kg and in shoot from 3.05 to 14.70 mg/kg, with the maximum concentrations found in the root of *Herniaria cinerea* and shoots of *Asphodelus tenuifolius*. The concentrations of Cr in both roots and shoots of *Chrysanthemum coronarium* and *Herniaria cinerea*, in roots of *Herniaria cinerea* and *Festuca ovina* and in shoots of *Plantago afra* exceeded the phytotoxic level [14]. The average concentrations of Co in different parts of the studied plants ranged from 0.51 to 2.02 mg/kg. The highest concentration of Co was observed in roots of *Festuca ovina* (2.02 mg/kg) and shoots of *Echium plantagineum* (<0.68 mg/kg). Co concentrations in most of the studied plants greatly exceeded the phytotoxic level [23], which was likely related to elevated Co in phosphate wastes of the Kettara mining site. The *Aizoon hispanicum*, *Emex spinosa, Plantago afra*, and *Spergularia rubra* accumulated Co in both roots and shoots. The concentration of Co was higher in roots compared to shoots except in *Emex spinosa*, and *Hirschfeldia incana*, suggesting a restrictive translocation to the shoots. To a lesser extent, the concentration of Ni and Zn presented a significant difference in plants and were slightly elevated than the minimum level for some plants’ species (*p* ≤ 0.05). The zinc concentrations ranged from 43.91 mg/kg to as high as 182.88 mg/kg in roots and from 22.73 to 211.97 mg/kg in shoots (*p* ≤ 0.05), with the highest value in roots of *Plantago afra* and shoots of *Spergularia rubra*. Regarding the phytotoxic levels of Zn (100–300 mg/kg) [14], five plant species accumulated excessive amount of Zn in roots: *Aizoon hispanicum*, *Plantago afra, Herniaria cinerea, Spergularia rubra and Reichardia tingitana* and three others in their shoots: *Hirschfeldia incana, Reichardia tingitana* and *Spergularia rubra*. The average concentration of Ni in roots and shoots of plant species ranged from 1.98 to 45.93 mg/kg with the maximum in roots of *Herniaria cinerea* and shoots of *Asphodelus tenuifolius*.

In contrast, As and Pb concentrations in plants biomass were significantly low (*p* ≤ 0.05) among the studied plant species, ranging from 0.54 to 15.98 for As and from non-detectable trace to 55.96 mg/kg for Pb. Compared with the content of trace elements in general plants [14], As and Pb concentrations in the studied species are less than the excessive phytotoxic level. As a total, Cd and Cu concentrations in plants species were low, ranging from non-detectable trace to 28.72 mg/kg for Cd and from non-detectable trace to 111.22 mg/kg for Cu. 

The concentrations of Cd in plants species were less than the excessive phytotoxic level except the *Plantago afra* with 28.72 mg/kg in the roots. The Cu concentration was significantly higher than toxicity levels, in the statistical sense (*p* ≤ 0.05) in the aerial and root parts of *Plantago afra* (60.56 and 32.97 mg/kg, respectively), and in the root part of *Herniaria cinerea* (111.22 mg/kg) and *Festuca ovina* (48.23 mg/kg). Concentration of Cu in all other species was below the excessive phytotoxic level. 

The present study reveals that many spontaneous plant species can colonize the phosphate wastes. A total of 13 plant species belonging to 10 families were collected. Previous studies [14,24] indicated that the phytotoxic concentrations of metals in all spontaneous plants tended to be much higher in roots than in shoots and attributed this to possible immobilization of heavy metal in the vacuoles of the root cells [25], thus rendering them less toxic. In the same context, our analysis reveals that all species had a significantly higher concentration of Cr (*p* ≤ 0.05) in the roots than in the shoots except for *Eryngium ilicifolium, Hirschfeldia incana, Plantago afra* and *Sperularia rubra* which had a higher concentration of Cr in the shoots than in roots. For the *Emex pinosa, Festuca ovina* and *Herniaria cinerea*, their roots had a higher concentration of Cu in the roots than in shoots, and the same for *Plantago afra, Asphodelus tenuifolius* and *Festuca ovina*, their roots had a high concentration in Cd. It is interesting to note that the root system had a capacity of metal uptake and inhibited excessive accumulation in shoots. This is probably a natural response of plants to high level of toxicity.

The Cr is not an essential element but there are reports of its stimulation of plant growth in low concentrations in solution culture or when added directly to soil. However these observations still remain unexplained and require further investigations [26]. Co is a transition metal not essential for plants, while Cu is an essential metal for plants growth and has a necessary role in enzyme composition for protein synthesis and photosynthesis [21,27,28,29,30,31]. Generally, metal concentration in plants is a major concern for a revegetation of a metal-contaminated site. According to the international standards [32], in the present study, we found that the concentrations of all metals in the aboveground parts of the sampled species were below the regulatory limits of the U.S. domestic animal toxicity standards for cattle grazing (Cd < 10 mg/kg, Zn < 500 mg/kg, Pb < 100 mg/kg). According to Baker (1981) [33], plants have two tolerance strategies for metals, either by exclusion or by accumulation of metals. In this study, high concentration of certain metals in the plant species indicated accumulation to these heavy metals, as part of its tolerance strategy. Regarding the stabilization of metals in contaminated areas, Wei et al. (2005) [34] argue that metal exclusion is preferable because plants have a high tolerance to metals and low translocation to aboveground tissues, which reduces the risk of metals transfer to the ecosystem through plants’ aboveground tissues and contaminating of the food chain.

### 2.4. Translocation Factor and Bioaccumulation Factor

Table 5 shows the bioconcentration (BCF) and translocation (TF) factors of the studied plant species and metals. All plant species presented a BCF < 1 for Cr and Cu. However, some of them had an ability to translocate Cr and Cu (TF > 1) to their aboveground parts. These included *Echium plantagineum, Eryngium ilicifolium, Hirschfeldia incana, Plantago afra, Reichardia tingitana* and *Spergularia rubra* for the translocation of Cr; and *Aizoon hispanicum, Asphodelus tenuifolius, Chrysanthemum coronarium, Eryngium ilicifolium, Plantago afra,* and *Spergularia rubra* for the translocation of Cu. Besides these plant species, all others restricted Cr and Cu accumulation in their aboveground biomass. All plant species showed a BCF < 1 for Cd, except for *Plantago afra* (BCF = 2.69). All plant species restricted Cd accumulation in their roots.

Five plant species only had an ability to transfer Cd to aboveground parts with a TF values significantly higher than 1 (*p* ≤ 0.05) (*Scolymus hispanicus, Emex spinosus, Echium plantagineum, Spergularia rubra* and *Aizoon hispanicum*).

Among the sampled plants, six species namely *Aizoon hispanicum, Asphodelus tenuifolius, Echium plantagineum, Festuca ovina, Herniaria cinerea*, and *Plantago afra* showed a BCF values significantly higher than 1 (*p* ≤ 0.05) and TF < 1 for Co, indicating that these species retained Co in their roots and limited its mobility from roots to shoots once absorbed by roots of plants. Hence, they could be labeled as good candidates for Co phytostabilization.

All plant species showed a low BCF for As, Pb and Ni except *Herniaria cinerea* which exhibited a remarkably high BCF, and a low TF level for these metals (Table 5). This species can be used for Pb, Ni and As phytostabilization. On the other hand, *Echium plantagineum, Hirschfeldia incana, Plantago afra, Scolymus hispanicus,* and *Spergularia rubra* showed a TF values significantly higher than 1 (*p* ≤ 0.05) for Pb and As. Among the sampled plants, three species were most suitable for Zn phytostabilization. It was *Aizoon hispanicum, Plantago afra* and *Herniaria cinerea* that showed a BCF values significantly higher than 1 (*p* ≤ 0.05) and TF < 1. However, four other species exhibited a high TF of Zn that reached 2.28 and 2.40 in *Echium plantagineum* and *Hirschfeldia incana*, respectively (Table 5).

Regarding to present results, Bioconcentration factor (BCF) of studied plant species were significantly higher for Co (*p* ≤ 0.05) followed by Zn, Cd, Pb, Ni, and As. Six species showed BCF values >1 for one, two, three or four metals: Cd, Co, As, Zn, Ni and Pb. *Herniaria cinerea* was appeared most efficient in bio-concentrating four metals in its roots (As, Co, Ni, Pb and Zn), and *Herniaria cinerea*, with its good potential to accumulate As, Co, Ni, Pb and Zn, can be proposed to be used in phytostabilization of these metals both in phosphate wastes covers as well as in Cd, Co and Cu contaminated sites in general. *Plantago afra* had a capacity to accumulate Cd, Co, and Zn in its roots, and can be used in Cd, Co, and Zn phytostabilization. It is known that most species of the genera *Plantago* emerged as able to accumulate large amounts of Zn [35].

*Aizoon hispanicum* had potential to accumulate Co and Zn and can thus be a good candidate for Co and Zn phytostabilization, whereas *Asphodelus tenuifolius, Festuca ovina, Spergularia rubra* and *Echium plantagineum* had an ability to accumulate only one metal (Co). These metal-tolerant species with significantly high BCF and low TF can be used for phytostabilization of the phosphates wastes cover of Kettara mining site.

### 2.5. Plant Species Candidate for Phytostabilization

**Figure 2:**
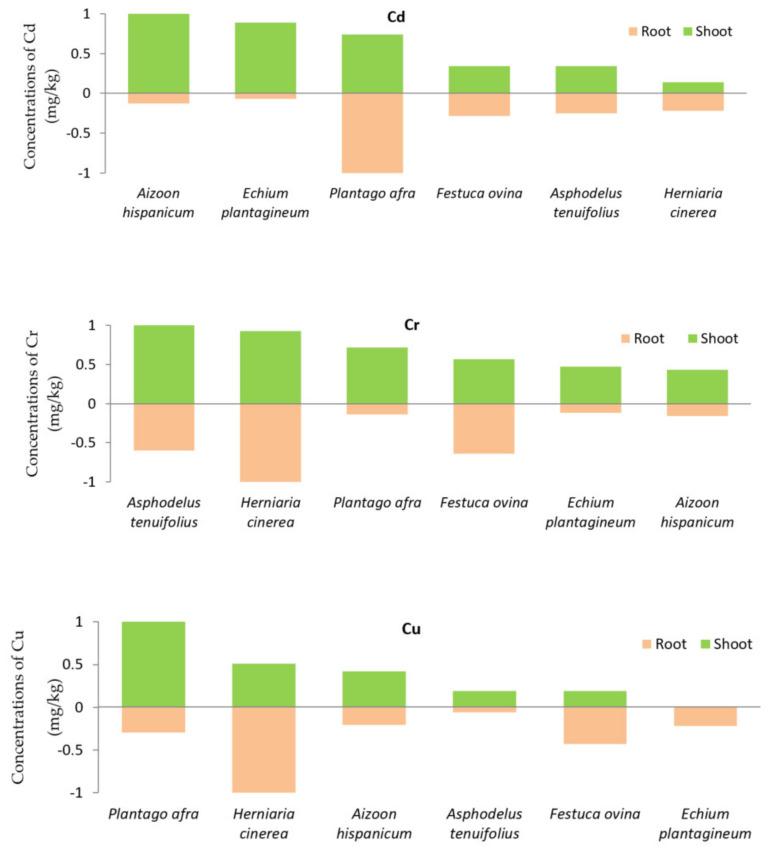
Candidate plants for the phytostabilization of phosphate wastes cover in increasing order of accumulation in shoots of Cd, Cr, and Cu metals. Root’s concentrations have been assigned negative values for plotting purpose.

In a phytostabilization technique for the remediation of contaminated sites, selection of appropriate plant species is essential. In the presence of large concentrations of metals, plants must have a low rate of translocation of metals from the roots to the shoots [36,37,38,39]. In the present study, the choice and evaluation of candidate plants for phytostabilization of phosphate wastes cover depend on the bioconcentration factor (BCF) and the translocation factor (TF) [40]. According to Yoon et al. (2006) [41], plant species with BCF greater than 1 and low TF could be considered candidate for phytostabilization, while plant species with TF higher than BCF could be used for phytoextraction. Based on this characterization and among the 13 studied plant species, *Hernaria cinerea* (As, Co, Ni, Pb and Zn), *Plantago afra* (Cd, Co, and Zn), *Aizoon hispanicum* (Co and Zn), *Echium plantagineum* (Co), *Asphodelus tenuifolius* (Co), and *Festuca ovina* (Co) had BCF greater than 1 and low TF. Moreover, these species have shallow roots that can spread horizontally to colonize phosphate coverings and develop a good coverage in a relatively short time. In addition, six of these species have accumulated much lower concentrations of metals and metalloids in their aboveground parts. The low translocation from the roots to the aboveground parts indicates that these species have useful characteristics for the phytostabilization of phosphate coverings. However, these six species exhibited large differences in their maximum concentration values. For ease of intercomparison, the metal concentrations in their shoots and roots were normalized by dividing both the shoots and roots concentrations by their respective maxima, and the roots concentrations have been assigned negative values for plotting purposes. The results are then sorted in ascending order using the shoots’ concentration. This normalization procedure was performed for the 6 plant species and the 3 metals that recorded concentration levels above and beyond the allowable range, namely Cd, Cu and Cr (Figure 2). This analysis reveals that the plant species with the least metal concentration in its aboveground part for a given heavy metal is the best candidate for phytostabilization of the substrate vis-a-vis that metal.

Our results also showed that a total of 6 species with a TF greater than 1 and a low BCF could be ideal for the phytoextraction of one or all metals in contaminated soils. It should be noted, however, that Aizoon hispanicum, Asphodelus tenuifolius, Echium plantagineum, Festuca ovina, and Plantago afra may have different phytostabilization and/or phytoextraction potential in different regions where the growing season and other environmental stresses may affect the performance and characteristics of the plant’s heavy metal accumulation. In addition, colonization of mine wastes by these metal-tolerant species would effectively reduce soil erosion and metal mobility, and would limit environmental degradation as well.

Specifically, we were able to identify the most one appropriate plant for phytostabilization of the phosphate limestone wastes for Cd (*Plantago afra*), and six plants for phytoextraction of the phosphate for each of Cu (*Aizoon hispanicum, Asphodelus tenuifolius, Chrysanthemum coronarium, Eryngium ilicifolium, Plantago afra,* and *Spergularia rubra*), and Cr (*Echium plontagineum, Eryngium ilicifolium, Hirschfeldia incana, Plantago afra, Reichardia tingitana*, and *Spergularia rubra*). The plant species are classified in increasing order from the least metal accumulating plant to the most metal accumulating plant in shoots, thus most suitable for the phytostabilization of the phosphate limestone wastes for the metal. These plants include *Aizoon hispanicum, Asphodelus tenuifolius, Echium plantagineum, Festuca ovina, Plantago afra,* and *Hernaria cinerea*. Our selection is based on metal accumulation in plant’s areal parts.

## 3. Materials and Methods

### 3.1. Study Area

**Figure 3:**
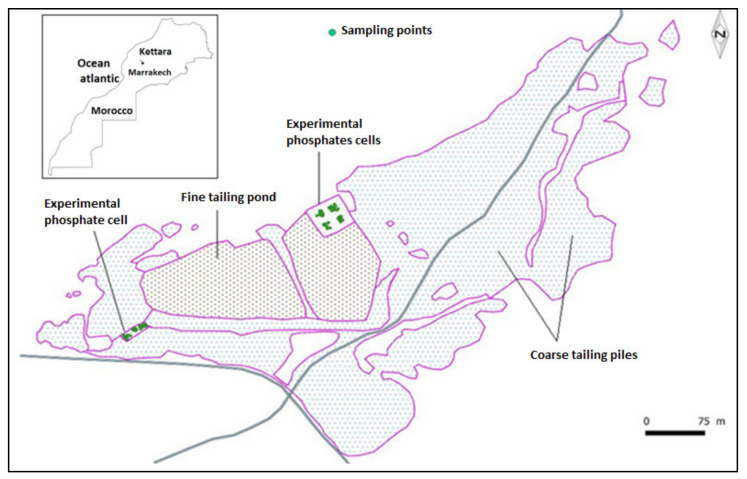
Geographical location of Kettara mine site and location of sampling area on experimental cells.

The study was carried out at the abandoned Kettara pyrrhotite mine located about 35 km north west of the city of Marrakech (Morocco). The mine is located exactly at 31°52′15′′ N and 8°10′31′′ W in the central mountains of Jebilet (Figure 3) and was exploited from 1964 to 1981. 

The Kettara mine includes the oldest and most exploited massive sulfides in this metallogenic area. During its 17 years of operation, the mine produced 5.2 Mt (1 Mt = 1 Million metric ton) of pyrrhotite concentrate, which contains on average 29% of sulfide [42]. Thanks to its role in the production of sulfuric acid and therefore in the development of the phosphate industry in Morocco, the mine has become one of the most important sites in Morocco’s 20th century mining industry history. More than 3 Mt of sulfide mine tailings, mainly rich in pyrrhotite, pyrite, arsenopyrite, chalcopyrite, galena and sphalerite, have been deposited over an area of approximately 16 ha. The mining tailings pond is surrounded by abandoned waste rocks which is mainly in the form of a dyke, the height of which is approximately 15 m. We also note the presence on the site, during dry periods, of precipitates of secondary minerals [4,5].

Although relatively small, about 1169 persons according to the latest census of 2014, the region’s population is located downstream from the mining site and is directly exposed to its toxic wastes. The area is characterized by a semi-arid climate with a monthly average temperature of 12 °C in January and 29 °C in July, an average total annual precipitation of 250 mm, and a potential annual evaporation of more than 2500 mm [43].

A rehabilitation project for the Kettara mining site has been implemented. The engineering reclamation approach aims to reduce the AMD leaching of sulfurous wastes by reducing water infiltration. A layer of fine alkaline waste rocks from the extraction of phosphates is scheduled to be placed on the abandoned mine tailings facility.

### 3.2. Collection of Plant and Rhizospheric Substrata Samples

For this study, 13 species of plants, growing naturally on the phosphates, as well as their rhizospheric soils were selected. Plant samples were collected from species dominant in abundance in the experimental phosphate cells (Figure 3). The sampling, consisting of 3 individuals from each species, was conducted during the months of May and June to ensure maximum number of drought-resistant species in the study region. For all soil samples, an area of 0.3 m^2^ of substrate was taken at a depth of 10 cm. The soil samples were homogenized and sieved to less than 2 mm [44].

The geographic coordinates of the sampling points were determined using a global positioning system (GPS) with the Lambert Northern Morocco cartographic projection and an accuracy of ±5 m.

### 3.3. Plant and Soil Samples Analysis

Plant sampling consisted of the selection of 3 individuals from each of the 13 species collected for a total of 39 samples. The sizes of the shoots and roots were measured for each sample. They were then separated, thoroughly washed with tap water, and rinsed with distilled water. Next, the samples were oven-dried at 80 °C for 3 consecutive days. The dried tissues were finally crushed into powder and mineralized.

Using a precision scale, we weighed several batches of 0.2 g of plant material that we placed in Polytetrafluoroethylene (PTFE) tubes. Two millimeters (ml) of concentrated nitric acid and 0.5 mL of concentrated hydrofluoric acid were then added to the plant material in each tube and mixed gently to moisten the entire sample. These digestion tubes were then closed, placed in the heating block and left overnight at room temperature. The next day, the tubes were heated 4 h at 110 °C while slightly open. At the end of this step, the lids are removed and 0.2 mL of hydrogen peroxide was added to the still hot solution; the addition of hydrogen peroxide is repeated twice while shaking. At the end of the reaction (about 10 s) the tubes were put back to heat. After that, 1 mL of concentrated nitric acid was added, the lids were replaced without being completely closed and we continued to heat for 4 h. The lids were then removed to allow the solution to evaporate to a final volume of about 2 mL. Then 2 mL of diluted nitric acid were added, the temperature of the block was lowered, and the heating continued for 5–10 min, making sure the solution did not start boiling. After cooling and eventually filtering of the residues, the mixture was transferred into a 10 mL tube, completing the volume up to 10 mL with distilled water [45].

The rhizosphere soil samples corresponding to each plant samples were dried at room temperature and then crushed to a particle size of less than 180 μm. The substrates were then digested according to the following procedure: (1) for 100 mg of soil, 2 mL of concentrated HNO_3_ were added; (2) the solution was then heated at 110 °C, and 3 mL of concentrated hydrofluoric acid were added after maintaining the solution for 15 h at 140 °C; (3) the solution was cooled to 110 °C and 2 mL of concentrated HNO_3_ were added; this operation is repeated three times for a total of 6 mL of HNO_3_. (4) Finally, 25 mL of HCl 2 M were added to the dry extracts before being analyzed [44]. The determination of total metals and metalloids concentration was determined by inductively coupled plasma atomic emission spectrometry (ICP-AES).

The mobile and mobilizable metal fractions were estimated separately using 10 millimolar (mM) of CaCl_2_ and 50 mM EDTA, respectively. Both solutions were neutral at pH 7. From each soil sample 2 g were suspended in 20 mL of CaCl_2_ for the mobile and 50 mM EDTA for the mobilizable, and stirred for 2 h. The suspensions were then centrifuged at 8000 rpm for 12 min and the metals’ mobile, and mobilizable concentrations were determined in the supernatants using ICP-AES [44].

For the pH measurement, 10 g of soil were suspended in 50 mL of distilled water. The mixture was stirred for 1 h using a magnetic stirrer and then decanted for 30 min. The pH was directly determined on the supernatant using a pH-meter [46].

### 3.4. Soil Pollution Index, Bioconcentration and Translocation Factors

The soil pollution index (PIs) is a criterion for assessing the toxicity of the soil. It identifies the multi-element contamination that results in an increase in metallic toxicity [47,48]. This index is calculated by averaging the ratios of the multi-element metal concentrations to their corresponding tolerable concentration level as defined by (1). A PIs greater than 1 corresponds to a polluted soil. 

The bioconcentration factor (BCF) is the capacity of a plant to accumulate a metal into its roots from the soil, and is computed by dividing the concentration of metal in the root by the concentration of total metal in the soil as shown in Equation (2) [49]. A BCF greater than 1 indicates a higher concentration in the root system. 

The translocation factor (TF) indicates the translocation of the heavy metal from the root to the shoot of plant, and is calculated by dividing the concentration of metal of the plant’s aerial tissues by the concentration of metal in the root as indicated by Equation (3) [49].

(1) $$\mathrm{PIs}=\frac{\sum \frac{\mathrm{Heavy}\;\mathrm{metal}\;\mathrm{concentration}\;\mathrm{in}\;\mathrm{soil}}{\mathrm{Tolerable}\;\mathrm{level}}}{\mathrm{Number}\;\mathrm{of}\;\mathrm{heavy}\;\mathrm{metals}}$$

(2) $$\mathrm{BCF}=\frac{\text{C-root}}{\text{C-soil}}$$

(3) $$\mathrm{TF}=\frac{\text{C-shoot}}{\text{C-root}}$$

### 3.5. Statistical Analysis

Data were analyzed using the Student–Newman–Keuls grouping test. The means and standard deviations (SD) were calculated. The Student–Newman–Keuls test consists of a stepwise multiple comparisons procedure used to identify statistically significant differences between sample means. In this study, the statistical significance was declared when *p* ≤ 0.05. The statistical analysis of metal concentrations was based on the analysis of variance (ANOVA) test using the SPSS software package version 21.

## 4. Conclusions

This study showed that many spontaneous plants species can colonize phosphate limestone wastes cover, which suggests that the soil is not completely contaminated and that plants do have some tolerance to heavy metal concentrations. The metal and metalloid concentrations in the soil revealed that three metals Cr, Cu and Cd exceeded by far their critical concentration values. The concentrations of metals and metalloids in the shoots and roots of the 13 plant species sampled in this study did not point to any hyperaccumulation since none of the concentrations of the 8 metals and metalloids in plant’s tissues reached the critical hyperaccumulation levels. However, based on the bioconcentration and translocation potential, we were able to identify plant species with the potential for phytostabilization and phytoextraction. In the restoration design of the Kettara mine, six species, *Plantago afra, Festuca ovina, Echium plantaginum, Aizoon hispanicum, Herniaria cinerea*, and *Asphodelus tenuifolius* appeared as good candidates for phytostabilization of the cover made with phosphate wastes to control AMD at the Kettara mine. Considering the most abundant trace metals in the phosphates cover (Cr, Cu, Cd), *Plantago afra* was identified as the most suitable for the phytostabilization strategy for Cd; and *Eryngium ilicifolium*, *Spergularia rubra,* and *Plantago afra* were identified as the most suitable for the phytoextraction strategy for Cu and Cr. The plant species selected in this work based on the transfer of trace elements have the advantage of growing naturally on phosphate rocks but are all annual species. They should be associated with perennial species to ensure plant cover throughout the year. Studies were carried out to select local species and to test, in pots culture and in the field, their growth potential on the phosphate substrate alone and in association with the spontaneous species selected. The results from the present study represent an important step forward in the definition of the rehabilitation scheme for the Kettara mining site. These results can be used for the rehabilitation of other waste rock dumps of phosphate mining which occupy large areas in Morocco.

## Figures and Tables

**Table 1:** Total concentrations, mobile and mobilizable fractions of metals and metalloids in the rhizospheric soils (mean ± SD, for three repetitions) in mg/kg, pH and the Soil Pollution Index (PIs) (unitless). The mobile and mobilizable data are expressed as percentages of the different fractions relative to the total in the phosphate wastes.

Parameters		As	Cd	Co	Cr	Cu	Ni	Pb	Zn	PIs	pH
Total	Mean	10.63 ± 1.91	10.67 ± 4.03	0.72 ± 0.16	138.04 ± 47.72	119.86 ± 59.89	25.07 ± 9.35	27.28 ± 15.42	104.70 ± 14.34	0.94	7.53
	Critical values ^a^	20.00	3.00	50.00	100.00	100.00	100.00	100.00	300.00		
Mobilizable EDTA	Mean	0.11 ± 0.06	0.22 ± 0.07	< 0.05	-	2.35 ± 0.64	-	0.24 ± 0.13	2.57 ± 1.05		
	Mean percent	0.011 ± 0.006	0.02 ± 0.01	< 0.07	-	0.02 ± 0.01	-	0.011 ± 0.07	0.03 ± 0.01		
Mobile CaCl2	Mean	< 0.05	< 0.02	< 0.05	-	< 0.02	-	< 0.06	< 0.01		
	Mean percent	< 0.005	< 0.002	< 0.07	-	< 0.002	-	< 0.003	< 0.0001		

^a^ The maximum allowable concentrations of trace elements in agricultural soils [14].

**Table 2:** List of plants collected in the phosphate wastes of the cover system tested at Kettara mine and their size measurements and life form.

Family	Species No	Species	Life-Form	Shoot System Size (cm)	Root System Size (cm)
*Aizoaceae*	1	*Aizoon hispanicum*	Annual	9.67 ± 4.04 ^a^	5.83 ± 2.25 ^a^
*Asphodelaceae*	2	*Asphodelus tenuifolius*	Annual/perennial	20.00 ± 5.00 ^a^	9.17 ± 2.47 ^a^
*Asteraceae*	3	*Chrysanthemum coronarium*	Annual	36.67 ± 15.28 ^ab^	14.83 ± 3.25 ^a^
	4	*Reichardia tingitana*	Annual	27.33 ± 6.43 ^ab^	8.33 ± 3.21 ^a^
	5	*Scolymus hispanicus*	Biennial/perennial	12.50 ± 1.32 ^ab^	9.67 ± 1.53 ^a^
*Boraginaceae*	6	*Echium plantagineum*	Annual	24.67 ± 17.79 ^ab^	14.33 ± 3.79 ^a^
*Polygonaceae*	7	*Emex spinosus*	Annual	22.50 ± 5.22 ^ab^	7.33 ± 2.08 ^a^
*Apiaceae*	8	*Eryngium ilicifolium*	Annual	4.33 ± 0.58 ^a^	10.43 ± 0.93 ^a^
*Brassicaceae*	9	*Hirschfeldia incana*	Annual	69.67 ± 21.50 ^b^	19 ± 11.27 ^a^
*Poaceae*	10	*Festuca ovina*	Annual	25.67 ± 5.51 ^ab^	12.33 ± 4.93 ^a^
*Caryophyllaceae*	11	*Herniaria cinerea*	Annual	11 ± 12.17 ^a^	6.77 ± 4.61 ^a^
*Plantaginaceae*	12	*Plantago afra*	Biennial/perennial	13.67 ± 2.93 ^a^	6.33 ± 1.76 ^a^
*Plumbaginaceae*	13	*Spergularia rubra*	Annual	14.50 ± 5.07 ^a^	5.33 ± 3.33 ^a^

The same letter within each column indicates no significant difference according to the Student–Newman–Keuls test at 95% confidence limit (*p* ≤ 0.05).

**Table 3:** Trace metals concentration in roots (mg/kg dry weight) of species growing in phosphate wastes cover at Kettara abandoned mine (each value represents the mean ± standard deviation of at least 3 repetitions).

Species	As	Cd	Co	Cr	Cu	Ni	Pb	Zn
*Aizoon hispanicum*	2.03 ± 1.22 ^a^	3.65 ± 1.43 ^a^	0.96 ± 0.54 ^a^	8.12 ± 2.65 ^ab^	23.77 ± 7.03 ^abc^	3.64 ± 1.56 ^ab^	22.55 ± 3.02 ^b^	162.86 ± 64.97 ^bc^
*Asphodelus tenuifolius*	1.30 ± 0.07 ^a^	7.10 ± 3.20 ^a^	1.30 ± 0.07 ^a^	30.01 ± 17.88 ^ab^	6.53 ± 3.81 ^a^	9.48 ± 4.04 ^ab^	12.51 ± 2.56 ^ab^	50.90 ± 12.98 ^a^
*Chrysanthemum coronarium*	2.37 ± 0.68 ^a^	5.36 ± 2.93 ^a^	0.69 ± 0.18 ^a^	46.95 ± 15.29 ^c^	9.94 ± 4.11 ^ab^	3.86 ± 0.66 ^ab^	14.22 ± 1.55 ^ab^	60.59 ± 7.71 ^a^
*Echium plantagineum*	1.85 ± 1.55 ^a^	nd	1.3 ± 0.74 ^a^	6.21 ± 1.42 ^ab^	24.28 ± 8.34 ^abc^	5.21 ± 0.21 ^ab^	nd	43.91 ± 25.7 ^a^
*Emex spinosa*	1 ± 0.44 ^a^	6.95 ± 1.78 ^a^	0.52 ± 0.02 ^a^	4.37 ± 0.28 ^ab^	30.63 ± 9 ^abc^	3.98 ± 1.59 ^ab^	2.83 ± 0.75 ^a^	70.62 ± 5.82 ^ab^
*Eryngium ilicifolium*	2.80 ± 0.99 ^a^	2.04 ± 1.61 ^a^	0.56 ± 0.05 ^a^	6.22 ± 1.47 ^ab^	4.34 ± 2.30 ^a^	3.89 ± 1.54 ^ab^	3.19 ± 0.58 ^a^	54.55 ± 22.25 ^a^
*Festuca ovina*	2.02 ± 0.61 ^a^	8.17 ± 3.41 ^a^	2.02 ± 0.61 ^a^	31.67 ± 10.93 ^c^	48.23 ± 24.63 ^c^	15.11 ± 6.54 ^c^	5.40 ± 0.25 ^a^	74.32 ± 10.77 ^ab^
*Herniaria cinerea*	15.98 ± 5.74 ^b^	6.18 ± 2.14 ^a^	<0.91 ^b^	49.77 ± 27.13 ^c^	111.22 ± 34.36 ^d^	45.93 ± 5.10 ^d^	55.96 ± 19.92 ^c^	127.92 ± 7.47 ^abc^
*Hirschfeldia incana*	<0.69 ^a^	4.59 ± 1.95 ^a^	<0.54 ^a^	3.08 ± 0.56 ^a^	5.1 ± 1.27 ^a^	3.14 ± 2.01 ^a^	0.88 ± 0.49 ^a^	44.19 ± 9.52 ^a^
*Plantago afra*	1.94 ± 0.16 ^a^	28.72 ± 19.15 ^b^	<2.25 ^a^	7 ± 1.67 ^ab^	32.97 ± 17.66 ^abc^	10.89 ± 4.55 ^bc^	5.33 ± 2.28 ^a^	182.88 ± 60.18 ^c^
*Reichardia tingitana*	0.84 ± 0.29 ^a^	nd	<0.63 ^a^	4.62 ± 1.08 ^ab^	nd	3.95 ± 0.32 ^ab^	0.85 ± 0.21 ^a^	120.97 ± 82.59 ^abc^
*Scolymus hispanicus*	0.73 ± 0.3 ^a^	4.73 ± 1.4 a	<0.51 ^a^	6.02 ± 3.78 ^ab^	27.91 ± 12.08 ^abc^	2.27 ± 0.01 ^a^	2.57 ± 0.63 ^a^	64.9 ± 31.5 ^ab^
*Spergularia rubra*	1.21 ± 0.54 ^a^	6.01 ± 0.35 ^a^	<0.7 ^a^	7.87 ± 4.54 ^ab^	9.59 ± 5.36 ^ab^	5.53 ± 2.45 ^ab^	<0.80 ^a^	151.50 ± 89.51 ^abc^
Toxicity levels ^1^	<2–80	6–10	0.4–several	0.2–1	20–30	10–50	0.6–28	100–300
The critical hyperaccumulating levels ^2^	>1000	>100	>1000	>1000	>1000	>1000	>1000	>10,000

The same letter within each column indicates no significant difference according to the Student–Newman–Keuls test at 95% confidence limit (*p* ≤ 0.05). ^1^ Critical toxicity level [14, 23]; ^2^ Threshold for hyper-accumulators [14,23].

**Table 4:** Trace metals concentration in shoots (mg/kg dry weight) of collected plants species growing in phosphate wastes cover in Kettara abandoned mine (each value represents the mean ± standard deviation of at least 3 repetitions).

Species	As	Cd	Co	Cr	Cu	Ni	Pb	Zn
*Aizoon hispanicum*	1.58 ± 0.74 ^ab^	7.59 ± 3.62 ^a^	<0.51 ^a^	6.34 ± 3.46 ^abc^	25.68 ± 4.52 ^a^	3.24 ± 0.33 ^ab^	12.89 ± 3.94 ^abc^	83.57 ± 12.82 ^ab^
*Asphodelus tenuifolius*	0.64 ± 0.1 ^a^	2.61 ± 0.07 ^a^	<0.57 ^a^	14.70 ± 6.74 ^c^	11.45 ± 8.02 ^a^	6.69 ± 1.52 ^ab^	4.72 ±0.32 ^a^	22.73 ± 5.79 ^ab^
*Chrysanthemumcoronarium*	<0.54 ^a^	3.40 ± 1.15 ^a^	<0.52 ^a^	4.19 ± 1.90 ^a^	10.83 ± 1.47 ^a^	3.97 ± 1.01 ^bc^	4.91 ± 2.02 ^a^	50.20 ± 8.01 ^c^
*Echium plantagineum*	2.52 ± 0.91 ^b^	6.75 ± 1.85 ^a^	<0.68 ^a^	6.94 ± 3.89 ^abc^	0.24 ± 0.07 ^a^	4.09 ± 1.81 ^a^	17.71 ± 4.74 ^bcd^	93.42 ± 25.63 ^ab^
*Emexspinosa*	<0.56 ^a^	8.75 ± 2.80 ^a^	<0.53 ^a^	3.05 ± 0.90 ^a^	9.29 ± 3.87 ^a^	2.19 ± 0.53 ^ab^	1.45 ± 0.41 ^abc^	44.95 ± 9.02 ^ab^
*Eryngium ilicifolium*	1.39 ± 0.62 ^ab^	5.11 ± 3.71 ^a^	<0.54 ^a^	9 ± 1.68 ^abc^	9.74 ± 0.06 ^a^	4.41 ± 0.23 ^a^	1.62 ± 0.73 ^a^	33.21 ± 13.19 ^ab^
*Festuca ovina*	<0.55 ^a^	2.56 ± 0.89 ^a^	<0.58 ^a^	8.33 ± 3.66 ^abc^	11.30 ± 5.12 ^a^	3.92 ± 0.14 ^ab^	4.79 ± 2.88 ^cd^	97.31 ± 50.53 ^ab^
*Herniaria cinerea*	1.58 ± 0.54 ^ab^	nd	<0.52 ^a^	13.74 ± 7.63 ^bc^	30.62 ± 13.54 ^a^	4.29 ± 1.67 ^a^	13.90 ± 7.49 ^cd^	99.84 ± 27.58 ^ab^
*Hirschfeldia incana*	1.27 ± 0.94 ^ab^	2.44 ± 0.69 ^a^	<0.57 ^a^	9.54 ± 2.67 ^abc^	<0.23 a	2.72 ± 1.1 ^ab^	11.68 ± 6.2 ^abc^	100.4 ± 47.11 ^ab^
*Plantago afra*	2.49 ± 1.39 ^b^	5.61 ± 3.52 ^a^	<0.54 ^a^	10.53 ± 0.63 ^abc^	60.56 ± 32.752 ^b^	4.24 ± 1.27 ^a^	7.27 ± 0.99 ^abc^	81.14 ± 13.46 ^ab^
*Reichardia tingitana*	<0.55 ^a^	nd	<0.55 ^a^	4.84 ± 1.39 ^a^	31.24 ± 20.34 ^a^	4.24 ± 0.64 ^ab^	3.01 ±2.05 ^d^	147.13 ± 107.06 ^ab^
*Scolymus hispanicus*	1.20 ± 0.98 ^ab^	5.20 ± 0.11 ^a^	<0.51 ^a^	5.31 ± 3.26 ^ab^	nd	1.98 ± 0.30 ^ab^	nd	82.91 ± 7.9 ^bc^
*Spergularia rubra*	2.34 ± 1.21 ^ab^	7.23 ± 1.71 ^a^	0.52 ± 0.01 ^a^	8.80 ± 0.19 ^abc^	16.43 ± 6.14 ^a^	6.21 ± 0.94 ^c^	2.22 ± 1.26 ^abc^	211.97 ± 90.41 ^ab^
Toxicity levels ^1^	<2–80	6–10	0.4–several	0.2–1	20–30	10–50	0.6–28	100–300
The critical hyperaccumulating levels ^2^	>1000	>100	>1000	>1000	>1000	>1000	>1000	>10,000

The same letter within each column indicates no significant difference according to the Student–Newman–Keuls test at 95% confidence limit (*p* ≤ 0.05); ^1^ Critical toxicity level [14,23]; ^2^ Threshold for hyper-accumulators [14,23].

**Table 5:** Bioconcentration factor (BCF) and translocation factor (TF) of trace elements in the sampled species from the phosphate wastes cover.

Species	BCF								TF							
	As	Cd	Co	Cr	Cu	Ni	Pb	Zn	As	Cd	Co	Cr	Cu	Ni	Pb	Zn
*Aizoon hispanicum*	0.19 ^a^	0.34 ^a^	1.34 ^a^	0.06 ^ab^	0.20 ^abc^	0.15 ^ab^	0.83 ^b^	1.56 ^bc^	0.80 ^a^	2.36 ^a^	0.64 ^cdef^	0.74 ^ab^	1.11 ^ab^	0.95 ^bcd^	0.56 ^a^	0.56 ^a^
*Asphodelus tenuifolius*	0.12 ^a^	0.67 ^a^	1.81 ^a^	0.22 ^abc^	0.05 ^ab^	0.38 ^abc^	0.46 ^ab^	0.49 ^a^	0.49 ^a^	0.42 ^a^	0.43 ^bcd^	0.60 ^ab^	1.88 ^ab^	0.77 ^abcd^	0.39 ^a^	0.45 ^a^
*Chrysanthemum coronarium*	0.22 ^a^	0.66 ^a^	0.96 ^a^	0.34 ^c^	0.08 ^abc^	0.15 ^ab^	0.52 ^ab^	0.58 ^ab^	0.24 ^a^	0.54 ^a^	0.78 ^defg^	0.09 ^a^	1.23 ^ab^	1.07 ^bcd^	0.34 ^a^	0.83 ^a^
*Echium plantagineum*	0.17 ^a^	0.12 ^a^	1.81 ^a^	0.04 ^ab^	0.20 ^abc^	0.21 ^ab^	0.07 ^a^	0.42 ^a^	2.06 ^a^	14.07 ^a^	0.55 ^bcde^	1.09 ^ab^	0.01 ^a^	0.78 ^abcd^	14.07 ^b^	2.28 ^b^
*Emex spinosa*	0.09 ^a^	0.65 ^a^	0.72 ^a^	0.03 ^ab^	0.26 ^abc^	0.16 ^ab^	0.10 ^a^	0.67 ^ab^	0.65 ^a^	1.26 ^a^	1.02 ^g^	0.70 ^ab^	0.35 ^ab^	0.59 ^abcd^	0.56 ^a^	0.65 ^a^
*Eryngium ilicifolium*	0.26 ^a^	0.19 ^a^	0.78 ^a^	0.05 ^ab^	0.04 ^a^	0.16 ^ab^	0.12 ^a^	0.52 ^a^	0.49 ^a^	6.60 ^ab^	0.96 ^fg^	1.49 ^b^	2.67 ^b^	1.26 ^d^	0.49 ^a^	0.62 ^a^
*Festuca ovina*	0.19 ^a^	0.77 ^a^	2.80 ^a^	0.23 ^bc^	0.40 ^c^	0.60 ^c^	0.20 ^a^	0.71 ^ab^	0.28 ^a^	0.34 ^a^	0.30 ^abc^	0.28 ^ab^	0.26 ^ab^	0.30 ^ab^	0.88 ^a^	1.29 ^ab^
*Herniaria cinerea*	1.50 ^b^	0.58 ^a^	16.91 ^b^	0.36 ^c^	0.93 ^d^	1.83 ^d^	2.05 ^c^	1.22 ^abc^	0.11 ^a^	0.21 ^a^	0.05 ^a^	0.28 ^ab^	0.30 ^ab^	0.09 ^a^	0.24 ^a^	0.78 ^a^
*Hirschfeldia incana*	0.06 ^a^	0.43 ^a^	0.74 ^a^	0.02 ^a^	0.04 ^ab^	0.13 ^ab^	0.03 ^a^	0.42 ^a^	2.08 ^a^	0.56 ^a^	1.08 ^g^	3.21 ^c^	0.05 ^a^	0.96 ^bcd^	16.99 ^b^	2.40 ^b^
*Plantago afra*	0.18 ^a^	2.69 ^b^	3.13 ^a^	0.05 ^ab^	0.28 ^abc^	0.43 ^bc^	0.20 ^a^	1.75 ^c^	1.33 ^a^	0.31 ^a^	0.25 ^ab^	1.57 ^b^	2.63 ^b^	0.41 ^abc^	1.42 ^a^	0.47 ^a^
*Reichardia tingitana*	0.08 ^a^	0.81 ^a^	0.87 ^a^	0.03 ^ab^	0.38 ^bc^	0.16 ^ab^	0.03 ^a^	1.16 ^abc^	0.67 ^a^	0.84 ^a^	0.88 ^efg^	1.04 ^ab^	0.76 ^ab^	1.07 ^bcd^	3.83 ^a^	1.20 ^ab^
*Scolymus hispanicus*	0.07 ^a^	0.44 ^a^	0.71 ^a^	0.04 ^ab^	0.23 ^abc^	0.09 ^a^	0.09 ^a^	0.62 ^ab^	1.95 ^a^	1.13 ^a^	1.00 ^fg^	0.88 ^ab^	0.44 ^ab^	0.87 ^bcd^	1.86 ^a^	1.41 ^ab^
*Spergularia rubra*	0.11 ^a^	0.57 ^a^	0.71 ^a^	0.06 ^ab^	0.08 ^abc^	0.22 ^ab^	0.03 ^a^	1.45 ^abc^	0.80 ^a^	2.36 ^a^	0.64 ^cdef^	0.74 ^ab^	1.11 ^ab^	0.95 ^bcd^	0.56 ^a^	0.56 ^a^

The same letter within each column indicates no significant difference according to the Student–Newman–Keuls test at 95% confidence limit (*p* ≤ 0.05).

## Data Availability

Not applicable.

## References

[1] Sonter L.J., Ali S.H., Watson J.E. (2018). Mining and biodiversity: Key issues and research needs in conservation science. R. Soc. B.

[2] More T.G., Rajput R.A., Bandela N.N. (2003). Impact of Heavy metals on DNA Contents in the whole body of fresh water Bivalve, *Lamellidens marginalis*. Pollut. Res..

[3] Wasi S., Tabrez S., Ahmad M. (2012). Toxicological effects of major environmental pollutants: An overview. Environ. Monit. Assess..

[4] Hakkou R., Benzaazoua M., Bussière B. (2008). Acid Mine Drainage at the Abandoned Kettara Mine (Morocco): 1. Environmental Characterization. Mine Water Environ..

[5] Hakkou R., Benzaazoua M., Bussière B. (2008). Acid Mine Drainage at the Abandoned Kettara Mine (Morocco): 2. Mine Waste Geochemical Behavior. Mine Water Environ..

[6] Khalil A., Hanich L., Bannari A., Zouhri L., Pourret O., Hakkou R. (2013). Assessment of soil contamination around an abandoned mine in a semi-arid environment using geochemistry and geostatistics: Pre-work of geochemical process modeling with numerical models. J. Geochem. Explor..

[7] Lghoul M., Kchikach A., Hakkou R., Zouhri L., Guerin R., Bendjoudi H., Teíxido T., Penã J.A., Enriqué L., Jaffal M. (2012). Etude géophysique et hydrogéologique du site minier abandonné de Kettara (région de Marrakech, Maroc): Contribution au projet de réhabilitation. Hydrol. Sci. J..

[8] Bossé B., Bussière B., Hakkou R., Maqsoud A., Benzaazoua M. (2013). Assessment of Phosphate Limestone Wastes as a Component of a Store-and-Release Cover in a Semiarid Climate. Mine Water Environ..

[9] Bossé B., Bussière B., Hakkou R., Maqsoud A., Benzaazoua M. (2015). Field experimental cells to assess hydrogeological behaviour of store-and-release covers made with phosphate mine waste. Can. Geotech. J..

[10] Bossé B., Bussière B., Maqsoud A., Hakkou R., Benzaazoua M. (2016). Hydrogeological behavior of a store-and-release cover: A comparison between field column tests and numerical predictions with or without hysteresis effects. Mine Water Environ..

[11] Knidiri J., Bussière B., Hakkou R., Bossé B., Maqsoud A., Benzaazoua M. (2017). Hydrogeological behaviour of an inclined store-and-release cover experimental cell made with phosphate mine wastes. Can. Geotech. J..

[12] Cooke J.A., Johnson M.S. (2002). Ecological restoration of land with particular reference to the mining of metals and industrial minerals: A review of theory and practice. Environ. Rev..

[13] Zine H., El Berkaoui M., El Adnani M., Hakkou R., Smouni A., Fahr M., Bouab N., El Faiz A., Ouhammou A. (2018). Screening for native plant species potential revegetation of phosphatic clay applied as a cover to abandoned Kettara mine tailings Marrakech, Morocco. SMETox J..

[14] Kabata-Pendias A. (2010). Trace Elements in Soils and Plants.

[15] Galfati I., Bilal E., Sassi A.B., Abdallah H., Zaïer A. (2011). Accumulation of heavy metals in native plants growing near the phosphate treatment industry, Tunisia. Carpathian J. Earth Environ. Sci..

[16] Li M.S., Luo Y.P., Su Z.Y. (2007). Heavy metal concentrations in soils and plant accumulation in a restored manganese mineland in Guangxi, South China. Environ. Pollut..

[17] Hakkou R., Benzaazoua M., Bussière B. (2009). Laboratory Evaluation of the Use of Alkaline Phosphate Wastes for the Control of Acidic Mine Drainage. Mine Water Environ..

[18] Miretzky P., Fernandez-Cirelli A. (2008). Phosphates for Pb immobilization in soils: A review. Environ. Chem. Lett..

[19] Fennane M., Ibn Tattou M., Ouyahya A., El Oulaidi J. (1999). Flore Pratique du Maroc.

[20] Fennane M., Ibn Tattou M., El Oualidi J. (2014). Flore Pratique du Maroc, Manuel de Détermination des Plantes Vasculaires.

[21] Yang S., Liang S., Yi L., Xu B., Cao J., Guo Y., Zhou Y. (2014). Heavy metal accumulation and phytostabilization potential of dominant plant species growing on manganese mine tailings. Front. Environ. Sci. Eng..

[22] Mikołajczak K., Kuczyńska A., Krajewski P., Sawikowska A., Surma M., Ogrodowicz P., Adamski T., Krystkowiak K., Górny A.G., Kempa M. (2017). Quantitative trait loci for plant height in Maresi × CamB barley population and their associations with yield-related traits under different water regimes. J. Appl. Genet..

[23] Krämer U. (2010). Metal Hyperaccumulation in Plants. Annu. Rev. Plant Biol..

[24] Chaney R.L. (1989). Toxic element accumulation in soils and crops: Protecting soil fertility and agricultural food-chains. Inorganic Contaminants in the Vadose Zone.

[25] Shanker A., Djanaguiraman M., Sudhagar R., Chandrashekar C., Pathmanabhan G. (2004). Differential antioxidative response of ascorbate glutathione pathway enzymes and metabolites to chromium speciation stress in green gram ((L.) R.Wilczek. cv CO 4) roots. Plant Sci..

[26] Bollard E.G. (1983). Involvement of unusual elements in plant growth and nutrition. Encycl. Plant Physiol. New Ser..

[27] Małecka A., Derba-Maceluch M., Kaczorowska K., Piechalak A., Tomaszewska B. (2009). Reactive oxygen species production and antioxidative defense system in pea root tissues treated with lead ions: Mitochondrial and peroxisomal level. Acta Physiol. Plant.

[28] Påhlsson A.-M.B. (1989). Toxicity of heavy metals (Zn, Cu, Cd, Pb) to vascular plants. Water Air Soil Pollut..

[29] Castagna A., Di Baccio D., Ranieri A.M., Sebastiani L., Tognetti R. (2014). Effects of combined ozone and cadmium stresses on leaf traits in two poplar clones. Environ. Sci. Pollut. Res..

[30] Bonanno G., Giudice R.L. (2010). Heavy metal bioaccumulation by the organs of *Phragmites australis* (common reed) and their potential use as contamination indicators. Ecol. Indic..

[31] Rodriguez E., da Conceição Santos M., Azevedo R., Correia C., Moutinho-Pereira J., de Oliveira J.M., Dias M.C. (2015). Photosynthesis light-independent reactions are sensitive biomarkers to monitor lead phytotoxicity in a Pb-tolerant Pisumsativum cultivar. Environ. Sci. Pollut. Res..

[32] NCR (National Research Council) (2005). Mineral Tolerance of Animals.

[33] Baker A.J.M. (1981). Accumulators and excluders-strategies in the response of plants to heavy metals. J. Plant Nutr..

[34] Wei S., Zhou Q., Wang X. (2005). Identification of weed plants excluding the uptake of heavy metals. Environ. Int..

[35] Gutiérrez-Ginés M.J., Pastor J., Hernández A.J. (2015). Heavy metals in native mediterranean grassland species growing at abandoned mine sites: Ecotoxicological assessment and phytoremediation of polluted soils. Heavy Metal Contamination of Soils.

[36] Wong M.H. (2003). Ecological restoration of mine degraded soils, with emphasis on metal contaminated soils. Chemosphere.

[37] Alvarenga P., Gonçalves A., Fernandes R., De Varennes A., Vallini G., Duarte E., Cunha-Queda A. (2008). Evaluation of composts and liming materials in the phytostabilization of a mine soil using perennial ryegrass. Sci. Total Environ..

[38] Tordoff G., Baker A., Willis A. (2000). Current approaches to the revegetation and reclamation of metalliferous mine wastes. Chemosphere.

[39] Mendez M.O., Maier R.M. (2007). Phytoremediation of mine tailings in temperate and arid environments. Rev. Environ. Sci. Bio/Technol..

[40] Wu Q., Wang S., Thangavel P., Li Q., Zheng H., Bai J., Qiu R. (2011). Phytostabilization Potential of *Jatropha Curcas* L. in Polymetallic Acid Mine Tailings. Int. J. Phytoremed..

[41] Yoon J., Cao X., Zhou Q., Ma L.Q. (2006). Accumulation of Pb, Cu, and Zn in native plants growing on a contaminated Florida site. Sci. Total Environ..

[42] SRM (Service Régional des Mines de Marrakech) (1983). Le Gisement de Sulfures Massifs de Kettara.

[43] ONEM (Observatoire Nationale de l’Environnement du Maroc) (1997). Monographie locale de l’environnement de la ville de Marrakech. Etude Réalisée Pour le Compte de la Wilaya de Marrakech.

[44] Marguí E., Queralt I., Carvalho M., Hidalgo M. (2005). Comparison of EDXRF and ICP-OES after microwave digestion for element determination in plant specimens from an abandoned mining area. Anal. Chim. Acta.

[45] Temminghoff E.J., Hoba V.J. (2004). Digestion with HNO3-H2O2-HF. Plant Analysis Procedures.

[46] Moreno J.L., Garcia C., Hernandez T., Pascual J.A. (1996). Transference of heavy metals from a calcareous soil amended with sewage-sludge compost to barley plants. Bioresour. Technol..

[47] Emoyan O., Ogban F., Akarah E. (2009). Evaluation of Heavy Metals Loading of River Ijana in Ekpan–Warri, Nigeria. J. Appl. Sci. Environ. Manag..

[48] Nishida H., Miyai M., Tada F., Suzuki S. (1982). Computation of the index of pollution caused by heavy metals in river sediment. Environ. Pollut. Ser. B Chem. Phys..

[49] Mishra T., Pandey V.C. (2019). Phytoremediation of red mud deposits through natural succession. Phytomanagement of Polluted Sites.

